# Surgical options in suprastomal collapse-induced severe airway obstruction

**DOI:** 10.1007/s00405-020-06339-3

**Published:** 2020-09-10

**Authors:** Serap Sahin Onder, A. Ishii, K. Sandu

**Affiliations:** 1 University of Health Science Umraniye Education and Research Hospital, Istanbul, Turkey; 2 Lausanne University Hospital, Lausanne, Switzerland

**Keywords:** Suprastomal collapse, Decannulation, Tracheostomy

## Abstract

**Purpose:**

A single institutions experience with various surgical options in the treatment of severe suprastomal collapse (SSC).

**Methods:**

The study included 18 tracheostomized children with SSC treated between January 2012 and December 2018. Data included: patient demography, initial airway lesions, comorbidities, indication and age at tracheostomy, prior airway surgery, stomal demography, type of surgery, postoperative management, complications and treatment outcomes.

**Results:**

Four techniques were used to correct SSC. The surgical choice was dependent on stoma demography and associated airway lesions. Excision was done in eight patients and rib cartilage augmentation in five. Three patients had single stage tracheal resection and anastomosis. Two patients received stomal rigidification and temporary placement of Montgomery T tube. Three patients with anterior rib graft augmentation required additional lateral tracheal wall rigidification. Three patients (two with cartilage augmentation, and one with stomal rigidification) developed minimal granulation tissue in the postoperative period. Complete SSC resolution was seen in all except two patients who had a partial response to the treatment. All patients were successful decannulated and are currently asymptomatic.

**Conclusion:**

Decannulation failures may be due to severe suprastomal collapse that could be either unique or associated with obstructing laryngotracheal lesions. Therefore, it is essential to select the most appropriate surgical treatment to obtain overall favorable outcomes.

**Electronic supplementary material:**

The online version of this article (10.1007/s00405-020-06339-3) contains supplementary material, which is available to authorized users.

## Introduction

A major complication of pediatric tracheostomy is decannulation failure, even after successful resolution of the reason why the tracheostomy was initially performed. Difficulties in decannulating a tracheostomized patient may be due to multiple causes like severe suprastomal collapse, tracheomalacia, peristomal granulation tissue and recurrence of laryngotracheal stenosis [1].

Suprastomal collapse (SSC) is an inward collapse of the anterior tracheal wall or cricoid cartilage above the stoma leading to dynamic airway obstruction during respiratory cycles. Chronic cricoid and upper tracheal compression by the cutaneous portion of the tracheostomy tube on the rapidly growing infant airway may cause this problem. The tracheal and cricoid cartilages in children are relatively pliable and hence can be easily bent by direct pressure, negative intra-tracheal pressure during inspiration and chronic local injury [2]. Severe SSC may be responsible for unsuccessful decannulation in almost one-fifth of young children with long-term tracheostomies [3].

The gold standard procedure to diagnose and characterize SSC is flexible transnasal laryngo-bronchoscopy in a spontaneously breathing patient. Although, a nearly complete obstruction of the tracheal lumen can be endoscopically observed, it is generally not a fixed stenosis and therefore the flexible bronchoscope can usually pass through it without trouble.

Several surgical techniques have been used to treat a unique and isolated SSC [1]. Various options include endoscopic excision, anterior cricotracheal suspension, autologous costal cartilage laryngotracheoplasty, tracheal resection and anastomosis, and external or/and internal splinting of the airway [3–6]. As mentioned earlier, SSC may be associated with other reasons of decannulation failures, and hence the stomal demography, associated laryngotracheal stenosis (LTS) and past interventions to treat LTS are important factors to consider in the overall patient treatment.

In this commentary, we retrospectively analyzed our units experience in managing SSC. We also propose an algorithm to select the type of surgical correction.

## Methods

This is a retrospective study of pediatric patients with suprastomal collapse, and treated in our unit between January 2012 and December 2018. Appropriate parental consent and hospital ethics committee approval (CER-VD 2019-02148) were obtained. Patient information was accessed through the hospital electronic database. The treatment charts were reviewed for patient demography, initial airway lesions, comorbidities, indication and age at tracheostomy, prior airway surgery, degree of SSC, type of surgery, postoperative management, treatment outcomes and complications.

This study includes 18 tracheostomized pediatric patients with documented SSC. Dynamic upper airway examination (video, supp. electronic material) was performed under spontaneous respiration using a small-for-age flexible bronchoscope (3.1 mm or 3.8 mm Olympus). While examining the tracheostoma, the cannula was temporarily removed and the stoma blocked on the skin with a sterile gauze without exerting excessive pressure that can physically occlude the trachea. A subglottic negative pressure was created using the suction channel of the bronchoscope and maintaining an optimal suction pressure (100–150 kPa). This allowed examining the stomal site, quantify its collapse and evaluate concomitant airway malacia. Additionally, a rigid laryngotracheoscopy was performed with 0°/30° Storz telescope.

To quantify the degree of stomal collapse, we adopted the classification system described by Pacheco et al. [3]. There were 14 patients with severe (> 75%) airway obstruction and 4 had moderate (50–75%) lumen obstruction. Endoscopy notes and videos were studied to determine additional airway pathologies.

Four surgical techniques were used to treat SSC depending on the stomal demography, concomitant presence of laryngotracheal stenosis (LTS) and if a prior intervention for LTS was performed. SSC corrective procedures were: (1) simple excision using either cold steel instruments or CO2 laser fiber, (2) single-stage tracheal resection and anastomosis (SS-TRA), (3) stomal rigidification using bio-resorbable mini plates, and (4) rib cartilage reconstruction.

**Fig. 1:**
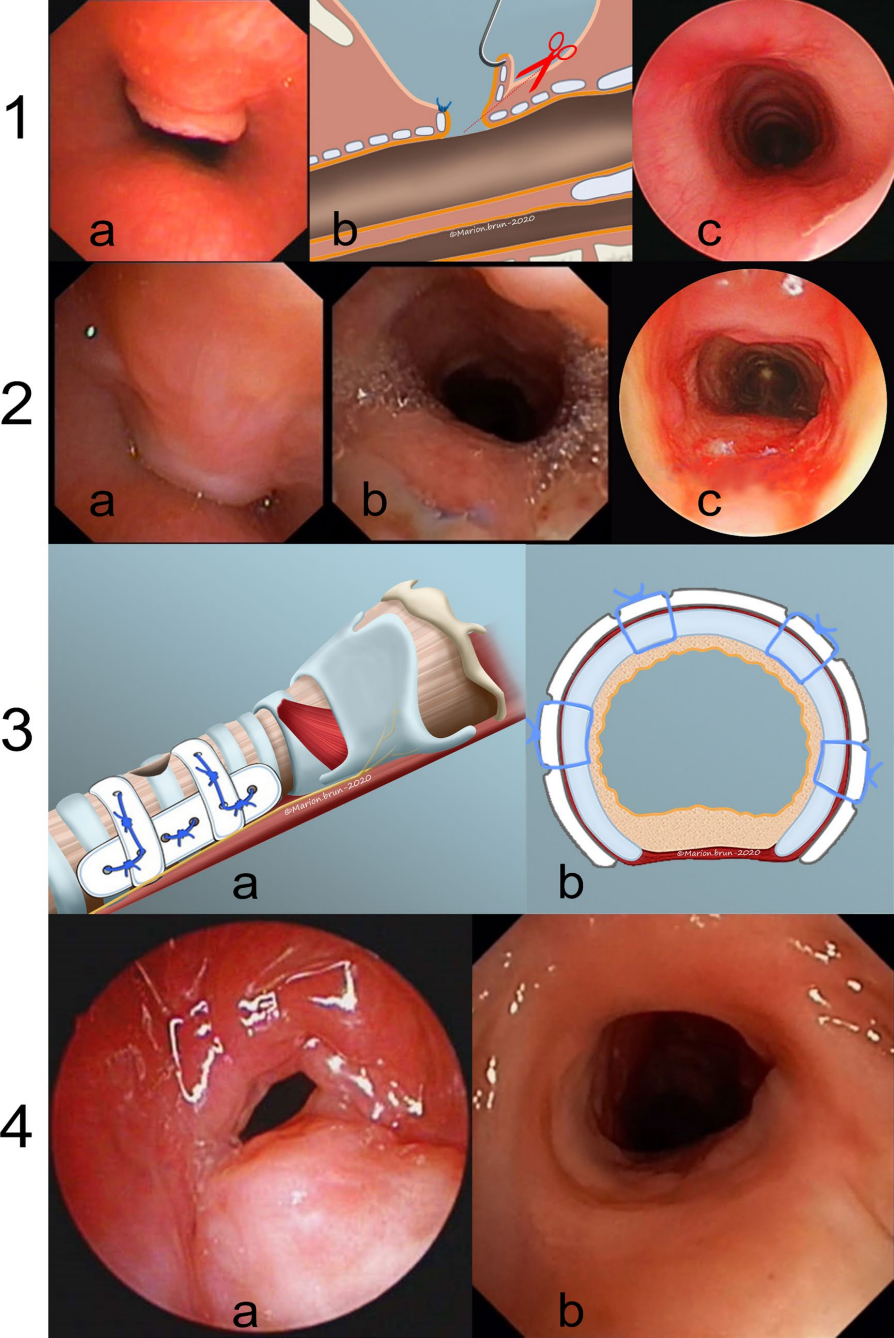
1. Local excision of suprastomal collapse. a Dynamic exam showing moderate degree of SS collapse. b SSC is hooked into the stoma and excised using fine forceps and scissors. c Dynamic exam view at 6 months. 2. Single stage segmental tracheal resection and anastomosis. a Severe peristomal SSC. b Dynamic exam view on day 5. c Postoperative view at 1 year. 3. Schema showing rigidification of the stoma. a Polydiaxanone (PD) micro plates are used to buttress the supra-, infra- and lateral margins of the stoma. b Axial view showing fixation of the plates on the supra- and infrastomal margins. 4. Stoma rigidification using PD plates. a Dynamic preoperative view showing stomal collapse and tracheal stenosis. b Dynamic exam showing an optimal postoperative result

Suprastomal collapse excision (Fig. 1a–c): The existing tracheostomy was used for induction of anesthesia. Suspension laryngoscopy was performed using Parsons Laryngoscope with a slide slot that allowed better surgical manipulation. Under trans-oral endoscopic guidance, a skin hook was passed through the tracheostoma to hook up the collapsing segment into the stoma and excised using fine tooth forceps and pointed curved scissors. If the stoma was small, contracted and prevented surgical manipulation through the stoma, a CO2 fiber laser (Omniguide; OmniGuide Inc. Massachusetts, 3-5 W) passed through a rigid bronchoscope was used to excise the SSC. The laser fiber was stuck on a broncho-telescope using Steristrips^R^ (ref. R1547, 3 M Health Care, USA). Telescopic observation validated the result.

Following SSC excision, the cannula was downsized and progressively capped (first with speaking and then with a full cap) during the daytime. With successful daytime tolerance of the fully capped cannula, a favorable polysomnography allowed extending the full capping into nighttime and then for full 24 h. The patient was monitored for respiratory symptoms. Two–three weeks later, the patient had another endoscopy (dynamic and rigid) to evaluate the stomal stability and reconfirm the airway safety. Following this, and during the same anesthesia, the tracheostoma was closed surgically similar to the technique in Ref. [7].

Single-stage tracheal resection and anastomosis (Fig. 1, 2a–c): This was performed in patients having severe circumferential stomal collapse with/without long cranio-caudal length. Two patients had additional lateral- and infra-stomal fibromas with complete tracheal obstruction. All patients were extubated uneventfully after 5–7 days.

Stomal rigidification (Fig. 1, 3a, b and 4a, b): This was performed in patients who had undergone prior cricotracheal resection and anastomosis and in whom further airway resection was not possible. Polydiaxanone Rapidsorb^R^ mini plates (DePuy Synthes, Johnson & Johnson) were used externally to splint the upper, lower and lateral walls of the tracheal cartilage around the stoma. The plates were fixed to the tracheal cartilage using 4.0 PDS sutures. Additional, internal splinting was done using Montgomery T tube (Hood Laboratories ST, ultra-smooth plus^R^) for 2–4 weeks and then replaced by a downsized cannula. Definitive stoma closure was done using strategy similar to after SSC excision.

Rib cartilage reconstruction: Large stomas (> 15 mm) and minor grade laryngotracheal stenosis with SSC required single-stage rib cartilage augmentation. The floppy SS segment was amputated and the anterior tracheal defect augmented using a rib graft. If necessary, the reconstruction was further reinforced by Rapidsorb^R^ mini-plates. Endotracheal intubation was required for 5–7 days.

All patients received amoxicillin clavulanic acid (40 mg/kg/day) for 5 days, and proton pump inhibitors (1 mg/kg/day) for 1 month postoperatively.

## Results

This is a 6-year-study of 18 pediatric patients diagnosed with suprastomal collapse causing moderate to severe airway obstruction. Table 1 shows the patients’ demography, indications of tracheostomy and degree of collapse. We did not investigate the risk factors causing SSC because majority of patients (*n* = 16; 89%) were referred from other centers and the tracheotomy was done by ORL, pediatric or general surgery units. Hence, precise information regarding the type of tracheal incision, cannula size used during the tracheotomy and then in the following months was difficult to obtain.

**Table 1:** Patient demography

	Patients (*n* = 18)	
Median age at tracheotomy	2 months	
İndication of tracheotomy		
Prolonged intubation	9	
Congenital BVCP	4	
CLW type 3	3	
Severe tracheomalacia	2	
Degree of collapse		
Moderate	2	
Severe	16	
Mean duration of tracheotomy time	26 months	
Mean time to decannulation following definitive surgery	73 days	

*BVCP* bilateral vocal cord palsy, *CLW* congenital laryngeal web

Eight girls and ten boys were included in the study. The age at tracheotomy ranged from 2 days to 16 years (mean: 15.4 months). The primary indications for tracheostomy were variable and prolonged intubation was the most common (*n* = 9). Other indications for tracheostomy were congenital bilateral vocal cord paralysis BVCP (*n* = 4), congenital laryngeal web CLW (*n* = 3) and severe congenital tracheomalacia (*n* = 2). Three patients had congenital heart defects, two were syndromic and developed severe grade III subglottic stenosis (SGS), three non-syndromic patients without comorbidities developed grade II SGS and 1 had severe bronchopulmonary dysplasia.

The SSC was first mentioned in the medical chart ranging from 2 months to 72 months (mean: 16.6 months) after the initial tracheostomy. The degree of collapse was severe in 16 patients and moderate in 2 patients.

Prior to treatment of SSC, seven patients had received treatment for their original airway pathology. Three out of four patients with BVCP received posterior rib grafts, and one had laser arytenoidectomy. Of the three patients with CLW, two had anterior and posterior grafts and one had only an anterior graft.

The mean age at surgical management of SSC was 41.9 months. Four techniques (Table 2) were used: Excision was done in eight patients (cold steel in five, laser in three); and rib cartilage augmentation in five patients (three patients had grade II subglottic stenosis and received anterior + posterior rib grafts in single stage). Two patients in this group had large stomas (18 mm, 21 mm, respectively) and hence received only an anterior graft. Three patients had single stage tracheal resection and anastomosis and both had four rings excised each, followed by cricotracheal anastomosis. Two patients with severe grade III SGS had undergone prior cricotracheal resection (done in two stages in the context of their syndrome) and received stomal rigidification by Rapidsorb mini plates together with concomitant Montgomery T tube placement. Three patients (one with large stoma, two with grade II SGS) required additional lateral tracheal wall rigidification in addition to anterior rib graft augmentation. Patients receiving stomal rigidification using bioresorbable plates had no recurrence in the follow-up period. Three patients (two received cartilage augmentation, and one had stomal rigidification) developed minimal granulation tissue in the postoperative period and responded to local excision and topical Mitomicin-C application. Complete SSC resolution was seen in all except two patients who had partial response to the treatment.

**Table 2:** Surgery for suprastomal collapse

Surgery for SSC	Patients (*n*)	Postop. complications	Resolution of SSC (complete/partial)	Decannulation (yes/no and TTD days)
Cold steel excision	5	–	C = 4; P = 1	Y, 28
Laser excision	3	–	C = 2; P = 1	Y, 32
Cartilage augmentation	5	Granulations (*n* = 2)	C	Y, 0
SS–TRA	3	–	C	Y, 0
Stomal rigidification	2	Minimal granulations (*n* = 1)	C	Y, 67

*SSC* Suprastomal collapse, *SS-TRA* single stage tracheal resection and anastomosis, *TTD* time to decannulation (mean)

The mean period of follow-up was 22.3 ± 4.5 months. All patients were successfully decannulated, currently have no respiratory symptoms and the last endoscopy showed no SSC recurrence. Patients following single stage tracheal resection and anastomosis and cartilage graft augmentation had their SSC and tracheostoma treated at the time of surgery (time to decannulation TTD: 0 day). However, those receiving stomal rigidification took the longest time (TTD: 67 days) to get decannulated.

## Discussion

Tracheostomy-related suprastomal collapse (SSC) is secondary to prolonged pressure on the cricoid and upper trachea by the cutaneous portion of the tracheostomy tube with subsequent chondritis and weakening of the tracheal cartilages [8]. Some degree of SSC is inevitable in children who have tracheostomy since a long time. Pediatric trachea seems to be more susceptible to this complication because of the less rigid nature of the younger tracheal cartilage or partial arrest of the normal tracheal growth rate. The type of trachea incision plays a role in SSC development. Anton-Pacheco et al. [3] reported an increased incidence of developing SSC in patients who had horizontal H-shape tracheal incision when compared to a vertical incision. A narrow tracheal opening made at the time of tracheostomy will cause excessive peri-cannular trauma to the cartilage during primary cannula insertion and later during its changes. SSC in its severe grade has been cited as one of the major causes for decannulation failure in pediatric patients [9].

There are many techniques detailing the correction of SSC. Some authors to overcome the suprastomal collapse have adopted the idea of external splinting of the trachea. In 1993, Azizkhan et al. developed the anterior cricotracheal suspension operation [2]. This is a single stage procedure aimed at elevating the suprastomal collapse by placing three or four extraluminal sutures between the weakened suprastomal segment and the overlying strap muscles. This would form a tough cicatricial tissue and provide a constant anterior traction on the collapsed area. Ochi described a surgical technique of hitching up the collapsed suprastomal trachea forward by suturing it anteriorly to the strap muscles on either side and achieve a greater airway diameter [10]. Bowe et al. achieved airway stiffening using bio-resorbable mini-plates for extraluminal rigidification of the weakened cartilage [5]. Decannulation was achieved in all their three pediatric patients with severe SSC. Froehlich et al. excised the suprastomal malacic tracheal wall and augmented the defect with costal cartilage graft. The authors concluded that this technique was efficient in patients with complete airway obstruction due to severe SSC [1]. Tawfik et al. reported excellent outcomes of their surgical technique in 12 patients with moderate to severe suprastomal collapse. The procedure included dissecting the tracheotomy tract up to the trachea, followed by its excision and then closing the stoma transversely by interrupted sutures [7].

Some authors adopted the idea of internal splinting of the trachea to manage the SSC. Park et al. used expandable stents to elevate the depressed suprastomal area [4]. Prescott et al. used a one-way speaking valve or simple plugging of the cannula to enhance the intraluminal pressure for mild and moderate collapse [10]. Endoscopic KTP laser in the excision of suprastomal collapse was proposed in less than 50% obstruction of the airway [6, 11].

In this report, we adopted four different surgical methods to correct the SSC that have been described in the literature. Our choice of surgery was influenced by: 1. stomal demography (size, associated peristomal malacia and obstructing concomitant lesions like granulomas and fibromas); 2. associated laryngotracheal stenosis (LTS), and 3. prior surgery for LTS. The most important point to critically analyze in cases of isolated and unique SSC is the stomal demography. Ours is a quaternary clinic managing complex airway problems and the referred patients do present with LTS recurrence and past interventions. If decannnulation is the ultimate goal, the treatment strategy must be aimed at concomitant treatment of SSC and the associated LT lesions.

We studied 18 patients in this report. The collapsing segment was excised endoscopically with cold steel instruments in five patients, and with CO2 laser in three patients. All these eight patients were decannulated successfully. Under suspension laryngoscopy and endoscopic guidance, a skin hook is passed through the tracheostoma and the floppy suprastomal segment hooked and delivered into the stoma. The SSC was excised using fine curved scissors. Endoscopic sinus surgery instruments like the sphenoid or Ostrum antral punch forceps can be used similarly. The endoscope passed trans-laryngeally provides an axial view of the SSC and allows its precise excision without damaging normal cartilage. The CO2 laser fiber was used when size of the stoma was too small to allow adequate hooking and delivery of the floppy segment into the stoma. The SSC is excised at the upper stomal margin.

Single-stage rib cartilage augmentation simultaneously treats suprastomal collapse associated with a large stoma and concomitant subglottic stenosis. We fixed the limit of a large stoma at 15 mm that corresponds to > 3 tracheal rings in infants and small children. All five patients in our series who had rib cartilage augmentation showed complete resolution of the collapse along with optimal treatment of their minor grade SGS. Patients with severe SSC, high grade severe SGS, no comorbidities and optimal vocal cord functions are treated best by single stage partial cricotracheal resection and anastomosis.

Three patients with severe circumferential stomal collapse and additional peri-stomal granulo-fibromatous lesions underwent single stage tracheal resection and cricotracheal anastomosis. In our opinion, unhealthy tracheostomas with severe irreversible collapsing cartilaginous and mucosal lesions are best treated by segmental resection and anastomosis allowing complete removal of the diseased trachea and anastomosing healthy adjacent laryngotracheal framework.

Patients who have undergone prior airway resection for LTS and later develop severe SSC may not be suitable to undergo a new airway resection and pose special problems. In two such patients, we performed amputation of the floppy segment, external stoma splinting with bioresorbable polydiaxanone mini-plates and concomitant Montgomery T tube placement. They were eventually decannulated. The tracheostomy cannula curvature induces a natural upward push of the suprastomal cartilage. A temporary T tube placement helps to maintain the stomal cartilage-to-skin angle at 90° until rigid stabilization of the stoma happens.

**Fig. 2:**
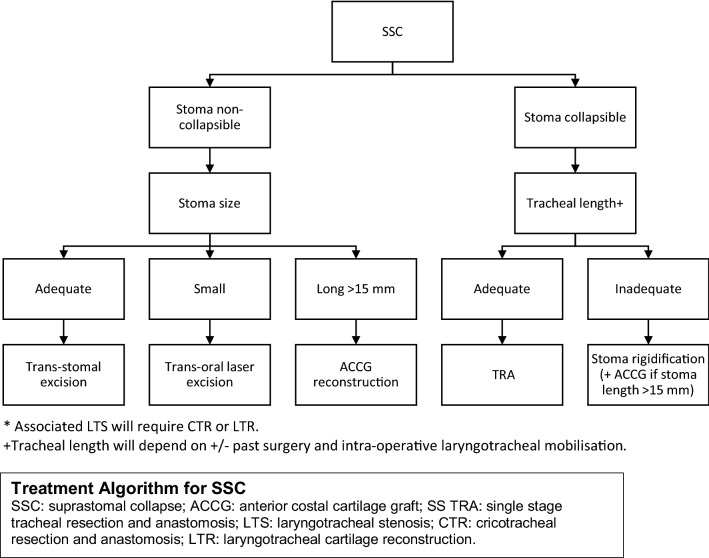
Treatment Algorithm for SSC

This commentary has several drawbacks. It is retrospective in nature and includes small heterogenous group of patients tracheostomised due to different etiologies. We feel that the choice of SSC corrective surgery must be tailor-made suiting each individual patient and therefore we propose an algorithm (Fig. 2)—validity of which would require larger multi-centric studies in homogenous patient groups.

Prevention of SSC development is critical. While performing a tracheotomy, various factors which could play a role in future SSC development must be kept in mind. Tracheal incision (the size should be slightly larger than the outer diameter of an age-appropriate cannula to avoid excessive stoma damage), primary maturation of the stoma, adequate nursing, regular follow up and close monitoring, timing of direct laryngoscopy and/or bronchoscopy evaluation while the tracheostomy is in place and decannulation technique are currently been studied in our unit and will be the topic of our future publication.

## Conclusion

The ultimate aim of treating patients with severe SSC is decannulation. Each patient with SSC may also have an associated airway pathology and hence it is crucial to make the correct choice for its surgical correction. This decision depends on the degree of suprastomal collapse, stoma characteristics, stability of the laryngotracheal complex, concomitant airway stenosis and past airway interventions.

## Electronic supplementary material

**Below is the link to the electronic supplementary material.**

**Supplementary material 1 (MOV 13958** **kb)**
